# Anesthetic Management of Laparoscopic Surgery in a Patient With an Uncomplicated Type B Aortic Dissection: A Case Report

**DOI:** 10.7759/cureus.105146

**Published:** 2026-03-13

**Authors:** Azhar Rehman, Alina Mahmood, Muhammad Saad Yousuf

**Affiliations:** 1 Anesthesiology, The Aga Khan University, Karachi, PAK; 2 Anesthesiology, Dow University of Health Sciences, Karachi, PAK

**Keywords:** aortic dissection, general anesthesia, glyceryl trinitrate, hemodynamic management, laparoscopic cholecystectomy

## Abstract

Aortic dissection is a rare but life-threatening condition requiring timely diagnosis and individualized management. Chronic type B aortic dissection is usually managed conservatively; however, anesthetic care during non-cardiac surgery poses unique challenges due to the risk of extension or rupture. We report the case of a 65-year-old hypertensive male with prior stroke who presented with symptomatic cholelithiasis. Preoperative imaging incidentally revealed a chronic Stanford type B aortic dissection extending from just distal to the left subclavian artery to the left common iliac artery. Cardiology and vascular surgery advised conservative management. He underwent laparoscopic cholecystectomy under general anesthesia with invasive monitoring. Induction was achieved with etomidate, morphine, and cisatracurium. Strict hemodynamic control was maintained with glyceryl trinitrate infusion, and intra-abdominal pressure was limited to 10 cm H₂O. The procedure lasted 54 minutes, was uneventful, and the patient recovered without complications. Anesthetic management of type B aortic dissection during laparoscopic surgery requires meticulous blood pressure and heart rate control to prevent dissection extension or rupture. Laparoscopic procedures increase intra-abdominal and systemic pressures, which can exacerbate aortic wall stress. In resource-limited settings, where short-acting agents may be unavailable, vigilant monitoring, multidisciplinary planning, and judicious use of available drugs such as morphine and vasodilators are essential to ensure patient safety. This case emphasizes the importance of perioperative vigilance, hemodynamic optimization, and multidisciplinary coordination in safely managing patients with chronic type B aortic dissection undergoing non-cardiac surgery.

## Introduction

Aortic dissection is defined as a tear in the innermost layer of the aortic wall, creating a true and false lumen. Aortic dissection is a life-threatening condition that must be diagnosed and treated without delay. The International Registry of Acute Aortic Dissection did a case series of 464 patients between 1996 and 1998 in which 62.3% of the patients had type A aortic dissection [1].

Aortic dissection is classified into two types according to the Stanford classification. Type A involves any part of the aorta proximal to the origin of the left subclavian artery, whereas type B involves the descending aorta and arises distal to the origin of the left subclavian artery [2]. Aortic dissection occurs due to weakening of the aortic wall, which can be caused by atherosclerosis, hypertension, aortic aneurysm, or iatrogenic dissection, and in younger patients may have some genetically mediated connective tissue disorder [3,4].

The first line of treatment for uncomplicated type B aortic dissection is medical therapy with periodic clinical and image surveillance [5]. In case of hemodynamic instability and complicated type B aortic dissection, urgent aortic repair is needed [6].

Anesthesia management in a patient with type B aortic dissection undergoing laparoscopic surgery is very high risk and can be very challenging, as there is a risk of extension of dissection or possible rupture leading to death, especially in resource-limited countries.

## Case presentation

**Figure 1:**
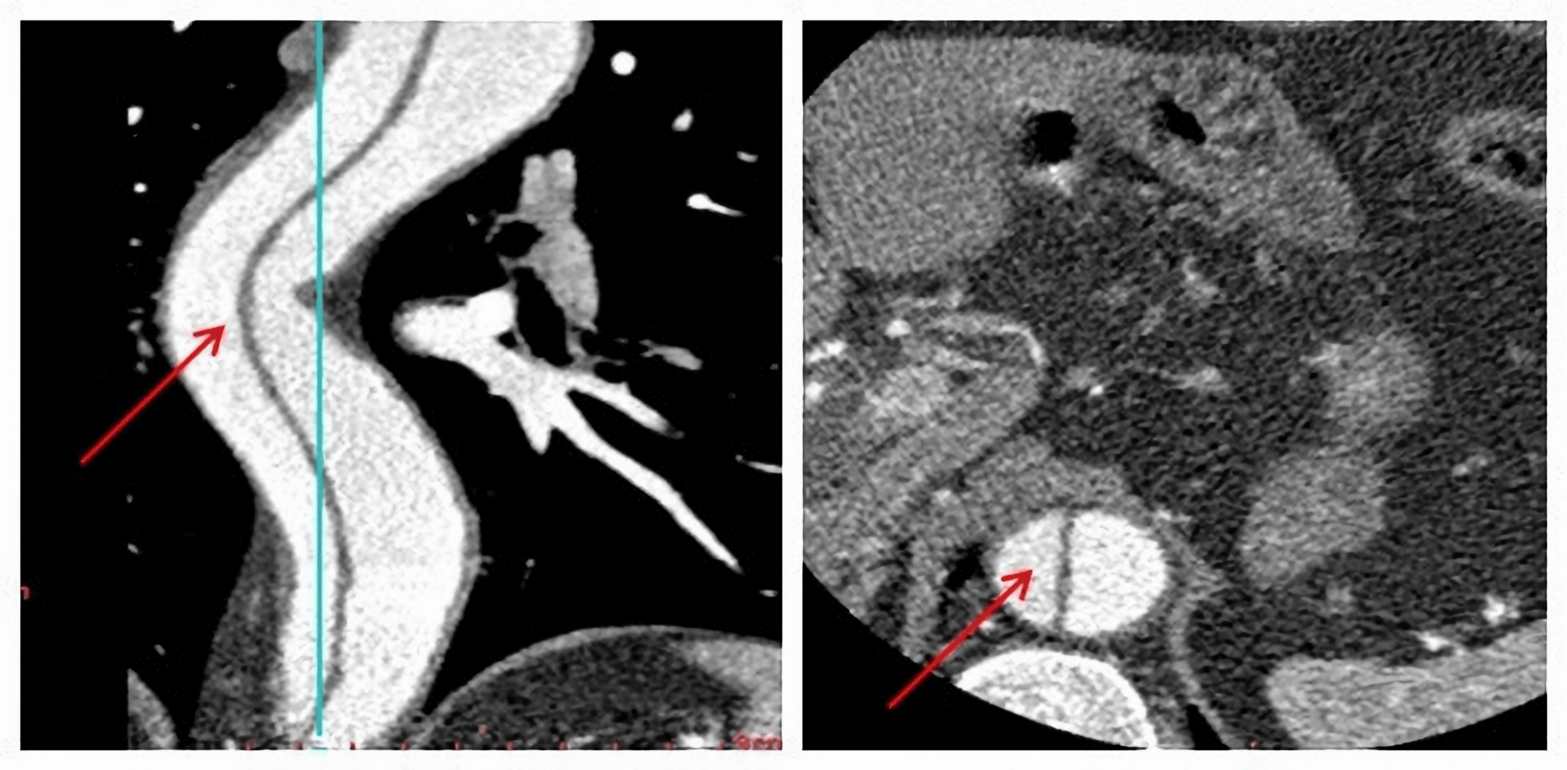
An angiogram showing apparent dissection as indicated by the red arrow, with axial and coronal views focused on the thoracic aorta

A 65-year-old gentleman with a history of hypertension and a stroke in 2016 with no residual weakness presented with a complaint of abdominal pain for the last four months. The patient was suspected of having cholelithiasis based on history and examination and was admitted for further advanced investigations and management, as he was being planned for laparoscopic cholecystectomy. Ultrasound of the abdomen showed a distended gallbladder with multiple stones. A computed tomography scan of the abdomen confirmed cholelithiasis and showed an incidental finding of type B aortic dissection. Subsequent computed tomography angiography indicated that the descending thoracic aorta has diffuse soft plaque throughout its course, with an aortic dissection flap starting just after the origin of the left subclavian artery, extending all the way to the ostium of the left common iliac artery (Figure 1). Descending aortic diameter was a maximum of 35 mm. Coronary angiography showed 60% ostial stenosis in the left anterior descending artery; the right coronary artery was dominant and medium-sized, showing the posterior descending artery branch with 70% ostial stenosis; the echocardiogram was normal. The electrocardiogram showed sinus bradycardia with a first-degree atrioventricular block and a heart rate of 51 beats per minute. All other biochemical and hematological investigations were normal. He was asymptomatic and denied having any history of chest pain, syncope, palpitation, or shortness of breath on moderate exertion. He was taking amlodipine/valsartan, a statin, and aspirin.

Cardiology and vascular surgery were taken on board, and they decided to manage the patient conservatively. He was then planned for laparoscopic cholecystectomy under general anesthesia; high-risk consent was taken. On the day of the procedure, the patient was premedicated with a tablet of midazolam, 7.5 mg, and he took his routine medications. In the operating room, monitors were applied, which included a three-lead electrocardiogram, non-invasive blood pressure, and pulse oximetry. In addition to that, an invasive arterial line was placed in the right radial artery, and a central venous catheter (CVC) was placed via the right internal jugular vein. Lignocaine 1.5 mg/kg was given to blunt the intubation response. Patient was induced with morphine 7 mg (due to unavailability of short-acting opioid, morphine was the only choice), etomidate 20 mg, and cisatracurium 12 mg; patient was intubated without any noticeable hemodynamic changes. Usually, pneumoperitoneum increases the intra-abdominal pressure, which in turn increases the systemic vascular resistance, so the surgical team was requested to keep the intrabdominal pressure around 10 cm of H₂O. It is essential to maintain the blood pressure of the patient within the normal range perioperatively to avoid complications. Therefore, after induction of anesthesia, glyceryl trinitrate infusion was started via CVC at the rate of 0.2 to 0.7 mcg/kg/min. Surgery took 54 minutes, and the patient remained stable. He was extubated safely and shifted to the post-anesthesia care unit for postoperative care and management. In the post-anesthesia care unit, he remained stable; therefore, he was shifted to the ward and then discharged home after a brief stay of one and a half days. The case has been reported in line with the Surgical CAse REport (SCARE) criteria [7].

## Discussion

Due to the ignorance of early signs and symptoms and the lack of early diagnosis and limited treatment in Pakistan, the perioperative mortality of aortic dissection is quite high; according to a study, it is reported as high as 35% [8]. Local data from one hospital showed only seven cases diagnosed in 15 years [9]. Its outcome is frequently fatal, and many patients with aortic dissection die before diagnosis. Even in this case, there was an incidental finding of chronic type B aortic dissection, which was identified during the patient's workup for gallstones. Although the incidence is very low, his case illustrates the importance of considering aortic dissection as one of the differentials when a chronic hypertensive patient with signs and symptoms of abdominal or chest pain presents in the emergency room. Anesthesia management of such patients can be testing throughout the perioperative period, in which a patient experiences the maximum catecholamine surge that could lead to a ruptured aorta. Laparoscopic surgery has become a standard of care for many abdominal surgical procedures with smaller incisions, a decreased perioperative stress response, minimal postoperative pain, and a quick recovery as compared to open surgery. During laparoscopic surgery, insufflation of carbon dioxide leads to a rise in intra-abdominal pressure, which increases the mean arterial pressure, systemic vascular resistance, and central venous pressure with a decrease in cardiac output and stroke volume [10]. These cardiovascular effects can be detrimental and can lead to end-organ ischemia, rapid expansion of dissection, aneurysmal degeneration of the aortic wall, expanding hematoma, or rupture. Hence, the major aim is to manage sympathetic responses to laryngoscopy, surgical manipulation, and incision. There are a number of studies focusing on perioperative hemodynamic management, but unfortunately, we lack short-acting and more sophisticated medications such as esmolol, remifentanil, fentanyl, etc. Weigang et al. state that, in patients with aortic dissection, pain relief and systolic blood pressure of 100 to 120 mm Hg can be achieved by administering morphine and beta-blockers in combination with vasodilators [11]. In this case, we used morphine for perioperative pain control and a glyceryl trinitrate infusion for the control of blood pressure. Throughout the procedure, the anesthesia team remained very vigilant and in communication with the surgical team, which resulted in a successful outcome.

## Conclusions

While providing anesthesia, blood pressure control, and pain management are the cornerstones in preventing detrimental consequences. Even with limited resources, we can manage high-risk patients with vigilance, proper multidisciplinary planning, and use the available resources wisely. 
